# Effects of triple semicircular canal plugging on hearing in patients with Meniere’s disease: A systematic review and meta-analysis

**DOI:** 10.1371/journal.pone.0314348

**Published:** 2024-12-05

**Authors:** Jia quan Zhu, Li li Kang, Xiao dong Wen, Jing Xiao, Yu jie Li, Rong Li, Hong jun Liu, Hong juan Wang, Fu yu Qiao, Dongbao Yang, Bin Xiang

**Affiliations:** 1 Department of Pharmacy, Fengdu General Hospital, Chongqing, China; 2 Department of Tumor Hematology, Fengdu General Hospital, Chongqing, China; 3 Department of Otolaryngology, Fengdu General Hospital, Chongqing, China; 4 Department of Administration, Fengdu General Hospital, Chongqing, China; UFPE: Universidade Federal de Pernambuco, BRAZIL

## Abstract

**Introduction:**

Triple semicircular canal plugging is effective in controlling vertigo in patients with Meniere’s disease, however, whether the rate of causing hearing loss during treatment is still not uniform. This study aimed to evaluate the effects of Triple semicircular canal plugging (TSCP) on hearing in Meniere’s disease (MD) patients.

**Methods:**

Databases Reviewed were PubMed, EMBASE, Scopus, Clinical Trials, Web of Science, Cochrane Library, CNKI, Wanfang, and VIP. The study reported on the duration, follow-up time, hearing loss, and vertigo control outcomes in MD patients evaluated by TSCP. Stata 17 software was used for data analysis and assessing the risk of bias.

**Results:**

This meta-analysis included 367 MD patients from 7 studies. TSCP efficiently alleviated vertigo with a rate of 99% (95% CI: 0.97, 1.00) and had a hearing loss rate of 22% (95% CI: 0.16, 0.28). Subgroup analysis revealed that for hearing loss, the proportion of patients with disease duration more than or less than 12 months was 14% (95% CI: 0.05, 0.26) and 24% (95% CI: 0.19, 0.29), respectively. For vertigo control, the rates were 100% (95% CI: 0.96, 1.00) and 99% (95% CI: 0.97, 1.00), respectively. For hearing loss, the follow-up time of MD patients more than or less than 24 months was 20% (95% CI: 0.07, 0.38) and 23% (95% CI: 0.18, 0.29), respectively. For vertigo control, the rates were 99% (95% CI: 0.95, 1.00) and 99% (95% CI: 0.97, 1.00), respectively. The duration of the disease and follow-up time had no significant impact on hearing loss and vertigo control rates.

**Conclusions:**

TSCP was efficient for vertigo control but with a risk of hearing loss. It could be used as a surgical method for hearing preservation in advanced MD patients.

## Introduction

Meniere’s disease (MD) is an inner ear disorder characterized by recurrent episodes of spontaneous vertigo, tinnitus, sensorineural hearing loss, and ear fullness [1]. It is one of the most common otogenic diseases around the world, with an incidence of 17–513 patients per 100,000 persons [2]. The pathogenic mechanisms of MD may include genetic, viral, immune, and anatomical factors, while endolymphatic exudative disorders are considered to be the main causative factor, but no consensus has been reached due to the complexity of the mechanisms involved in the development of MD [3]. Therefore, current treatment of MD mainly includes alleviation of acute vertigo and prevention of recurrence.

The choice of diagnosis and treatment usually depends on the different etiology and the severity of vertigo. The initial treatment is a symptomatic and comprehensive treatment based on drug therapy, which mainly reduces vertigo symptoms by regulating autonomic nerve function and improving inner ear microcirculation. About 85% of MD patients can be prevented from vertigo if they receive proper treatment and change lifestyles, but still 15% cannot control vertigo well [4]. For patients with refractory or recurrent vertigo who are ineffective to drug treatment, the corresponding surgical treatment should be selected according to the patient’s condition [5]. Tympanic injection of gentamicin or hormones and surgical procedures such as endolymphatic sac surgery (ESS), labyrinthectomy, and vestibular resection are optional for refractory MD. The indications are frequent, severe vertigo, and poor response to conservative treatment for more than 6 months.

Based on different aims, the surgical methods of vertigo can be divided into three categories: the surgical methods aiming at the possible causes and pathogenesis of vertigo, the surgical methods aiming at the obstruction of the vestibular conduction pathway, and the surgical methods for the reconstruction of vestibular and cochlear functions [6]. Semicircular canal plugging (SCP) is performed by opening a window in the semi-gauge bone canal and filling it with bone powder, fascia, biological glue, and other materials, or by laser blocking or blocking the flow of internal lymphatic fluid and the stimulation of otoconia on the ampulla crest receptor to control the vertigo. Hand techniques belonging to the second class [7].

SCP is initially used to treat benign paroxysmal positional vertigo [8] and later extends to other otogenic vertigo diseases. Yin et al. [9]. First advocated triple semicircular canal plugging (TSCP) as a surgical treatment for MD. Since then, multiple reports showed that TSCP in the treatment of MD could be well controlled [7, 10–12]. Compared with traditional vestibular surgery for Meniere’s disease, TSCP has the following advantages: It has a higher vertigo control rate and hearing retention rate, better protection of otolith function, shorter postoperative imbalance time, and faster establishment of mid-axis compensation, so it is widely used. The preservation of cochlear function is critical for SCP [13]. However, no study has characterized the effect of TSCP on the hearing of MD patients. Therefore, this meta-analysis depicted the postoperative hearing loss rate of MD patients after TSCP treatment, aiming to provide a better understanding of the effect of TSCP on hearing during MD treatment.

This systematic review and meta-analysis aim to evaluate the effects of TSCP on hearing outcomes in individuals diagnosed with MD. The primary objective is to determine the rate of hearing loss following TSCP treatment, utilizing the American Academy of Otolaryngology-Head and Neck Surgery (AO-HNS) guidelines for assessing changes in pure tone audiometry (PTA) thresholds. Hearing loss will be categorized based on the magnitude of the threshold increase: "hearing loss" is defined as an increase of 10 dB or more compared to preoperative levels, and "severe hearing loss" is defined as an increase of 20 dB or more. Additionally, the secondary objective is to assess vertigo control rates post-TSCP treatment, applying AO-HNS guidelines to categorize control outcomes into "complete vertigo control" (indicating no vertigo attacks) and "substantial vertigo control" (denoting a significant reduction in vertigo attacks). Through a comprehensive synthesis of available literature, this study aims to elucidate the impact of TSCP on hearing function in MD patients, contributing valuable insights into the efficacy of TSCP as a therapeutic intervention for managing MD-related symptoms, particularly its influence on hearing outcomes.

## Materials and methods

### Protocol and registration

This systematic review and meta-analysis were conducted following the guidelines of the PRISMA checklist [14] (S1 Table). According to the pre-designed scheme, systematic retrieval, conformity assessment, quality evaluation, data extraction, and data analysis were performed. The protocol for this review was registered and published in INPLASY (INPLASY 202340067).

### Study selection

We searched English databases including PubMed, Web of Science, Embase, Cochrane Library, Clinical Trials, and Scopus, and Chinese databases including Wanfang, CNKI, and VIP to identify relevant studies published as of December 2023. The search strategy was based on abstracts, titles, and keywords containing proper terms, which were presented in the S1 File. Studies were selected after we reviewed the title, abstract, and full text. The search strategy was based on abstracts, titles, and keywords containing proper terms, which were presented in the S1 File. Studies were selected after we reviewed the title, abstract, and full text. The searches were conducted simultaneously by four participants (ZJQ, KLL, WXD, and XJ), and the corresponding disagreements were resolved through consultation with a fifth senior (YDB) analyst.

### Eligibility criteria

Inclusion criteria were as follows: (a) TSCP treatment for MD with reported postoperative effects on hearing; (b) the study designs were randomized or non-randomized controlled study, cohort studies, retrospective studies, and case-control studies; (c) diagnosis of MD according to the 1995 American Criteria for Diagnosis of Meniere’s Disease in Otolaryngology, Head and Neck Surgery (AO-HNS) [15]; (d) all patients received conservative treatment for at least 6 months, including medication (betaestine 12 mg tid, hydrochlorothiazide 25 mg bid) and lifestyle changes, and tympanoid steroids; (e) postoperative follow-up time more than 6 months; (f) Data were expressed as a percentage or frequency; (g) hearing function was estimated using a pure tone audiometer and calculated by (a+b+c+d)/4 compiled following the 1995AO-HNS standard (a,b, c andd represented hearing levels at 0.5, 1, 2 and 3 kHZ, respectively). The worst hearing levels 6 months before or after surgery were compared. A change of 10 decibels (dB) or more was considered "better" or "worse" based on its tendency, and a change of less than 10 dB was considered "no change".

Exclusion criteria were as follows: (a) patients with normal hearing; (b) Meniere’s second operation; (c) republished literature; (d) postoperative hearing status was missed; (e) poor physical condition that cannot tolerate surgery; (f) studies were reviews, case reports, letters to the editor, conference papers, books, editorials, and notes; (g) articles on animals.

### Endpoints

The primary endpoint was defined as the rate of hearing loss, which was assessed using American Academy of Otolaryngology-Head and Neck Surgery (AO-HNS) guidelines. The change of mean pure tone auscultation (PTA) threshold was calculated and used for the classification of "hearing loss" (elevated 10 dB postoperative PTA threshold compared to preoperative one) or "severe hearing loss" (elevated 20 dB postoperative PTA threshold compared to preoperative one).

Secondary endpoints were vertigo control rates that were assessed using AO-HNS guidelines. Categories A and B of "complete vertigo control" and "substantial vertigo control", respectively were combined because Category B represented a substantial reduction (99 to 60 percent) in vertigo attacks, which was generally considered successful vertigo control.

### Data extraction

Studies that were screened for inclusion were blinded, with the concealment of all authors and institutions for each article that was included. The full texts of all relevant studies included in this process were retrieved and screened independently by the same reviewers. The study members whose data were extracted were not involved in the included studies. Two reviewers (LYJ and LR) independently screened the titles and abstracts according to the same selection criteria, and three reviewers extracted the data of potentially relevant studies based on predefined qualification criteria. Resolution of disagreements between the three reviewers (WHJ) was done based on the review of eligibility and discussion with a senior independent reviewer until a consensus was reached. After eliminating duplicate articles, we extracted data following the PRISMA guidelines: author, year of publication, total number of participants, number of people with hearing loss, number of people with vertigo control, sex ratio, age, duration of follow-up, duration of disease, and hearing before and after surgery. "NR" indicated missed data in the extracted article.For studies with missing important information (such as the number or rate of hearing loss, dizziness control, disease duration, follow-up time, etc.), data will be obtained by contacting the corresponding author. If data cannot be obtained, the study will be deleted and included.

### Risk of bias assessments

The quality of the seven articles included in this meta-analysis was assessed using the revised Newcastle- Ottawa Quality Assessment Scale (NOS) for nonrandomized studies [16]. Since our study was to explore the effect of TSCP on hearing loss and vertigo control in MD patients, the included studies were all one-arm observational studies. We used "*" to indicate that the research met the requirement of the corresponding dimension. The total score of the study quality ranged from 1 to 8, and scores ≥ 5 were considered to have a low risk of bias. Risk assessment was scored simultaneously by three independent authors (ZJQ, KLL, and WXD), with a fourth author (LHJ) participating in negotiations and reaching a consensus if there was a controversy.

### Statistical analysis

We used Stata 17 software to perform the meta-analysis. The random effects model was used to analyze. We conducted meta-analyses of pooled study outcomes only if the studies provided adequate data and did not have clinical heterogeneity. Otherwise, the results were presented as a narrative summary. The rate of hearing loss and vertigo control after TSCP treatment was calculated respectively. The effect size and 95% CI for each study were presented as forest maps. Heterogeneity was assessed using Cochran’s Q test (p < 0.05) and I^2^ statistics recommended in the Cochrane Manual when estimating hearing loss and vertigo control rates. The study heterogeneity was defined as low, medium, and high with I^2^ values of 25%-50%, 50%-75%, and > 75%, respectively [17]. To exclude heterogeneous impacts, we performed a subgroup analysis to explore the effects of follow-up time and disease duration time on hearing loss and vertigo control rates after TSCP treatment in 7 included studies. Moreover, a sensitivity analysis was adopted to assess the robustness and reliability of the pooled incidence. Egger’s regression model was used to quantify publication bias, and numerical methods were adopted to evaluate the impact of failure safety assessment bias [18, 19]. Specifically, it was performed by deleting studies one by one and conducting a meta-analysis after deletion. This cumulative analysis was widely used to estimate the impact of the largest studies on the pooled effect size. The p-value < 0.05 was considered statistically significant. If important information is not provided, the corresponding author will be contacted.

## Results

### Studies selection

**Fig 1:**
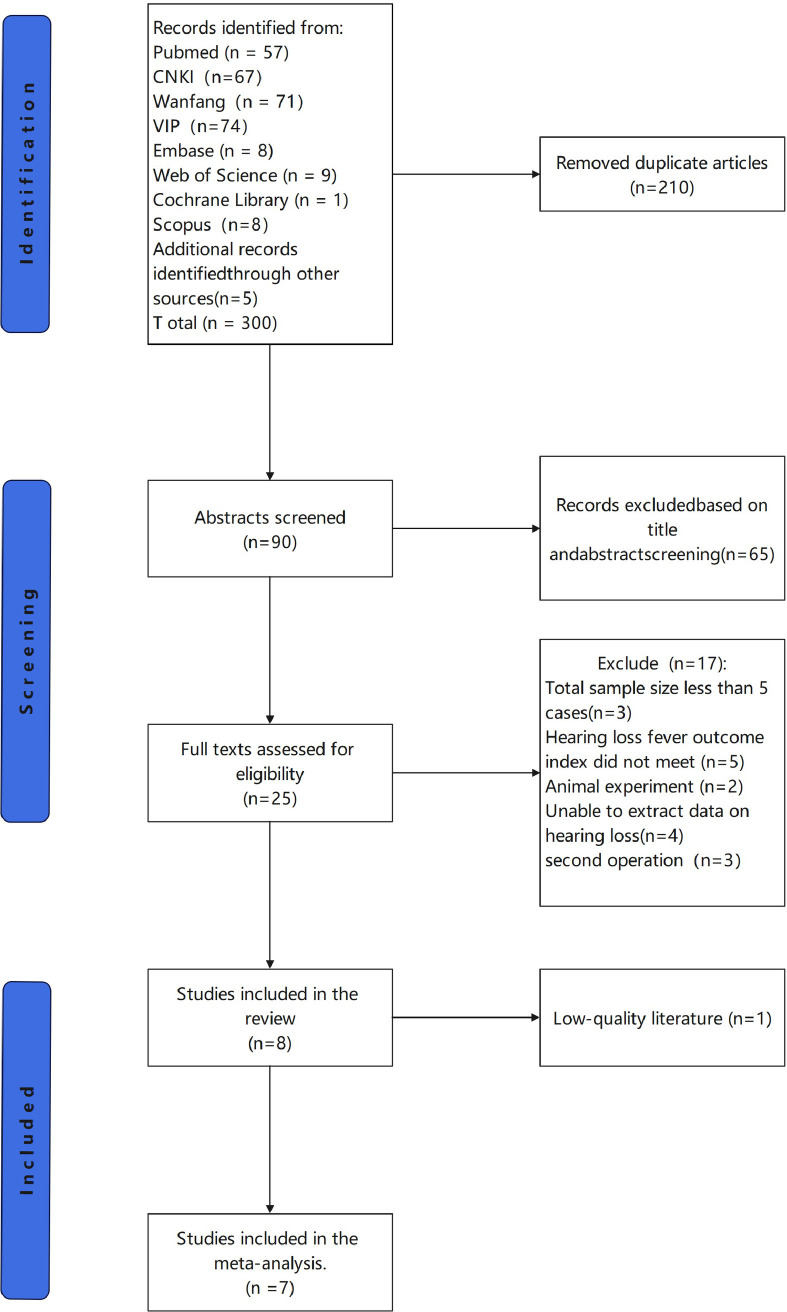
Research selection procedure based on the PRISMA flowchart.

Our system retrieved articles published as of December 31, 2022. A total of 295 studies were initially identified by the literature search and 5 additional articles were obtained in the references (S2 File), of which 210 duplicates were deleted and the remaining 90 articles were subsequently assessed for eligibility. After a review of article title and abstract, 25 of 65 articles were selected to read the full text. Among them, 15 articles were excluded because the number of patients with reported postoperative hearing loss did not meet the inclusion criteria. Then, the full text of 10 articles was reviewed and 3 papers were excluded because their definitions of hearing loss after TSCP surgery were not consistent with our inclusion criteria. Finally, the remaining 7 articles were included in this systematic review and meta-analysis (Fig 1).

### Characteristics of included articles

The characteristics of the included study are shown in Table 1 [7, 12, 20–24]. A total of 7 articles and 367 participants were enrolled. All participants were Chinese with 45% of male and 55% of female. They were all MD patients who previously received medication for more than 6 months and failed to control vertigo well. The duration time of MD ranged from 6 months to 120 months, and the mean postoperative follow-up time ranged from 10 months to 60 months. Hearing tests were conducted according to AO-HNS guidelines in all studies. The change in mean PTA threshold was calculated and classified as "hearing loss" or "severe hearing loss". Mean PTA thresholds for preoperative and postoperative hearing were reported in 2 studies. The baseline characteristics of all studies, including author and year of publication, total population, age, sex, number of people with hearing loss, duration of disease, and duration of follow-up were shown in Table 1. In addition, the assessment of bias risk showed that 7 studies had a low risk of biases (NOS score ≥ 5). The details of each NOS score are shown in the S2 Table.

**Table 1:** The characteristics of studies included in the systematic review and meta-analysis.

Study	Year	Study Design	Total	Age (year)	Male	Female	hearing loss	profound hearing loss	The control of vertigo	Pre-op PTA (dB)	Post-opPTA (dB)	Duration (month)	Follow-up (month)
Xuhui Liang [23]	2017	retrospective study	29	58.8±11.2	11	18	2	0	29	NR	NR	6	13
Zhaoming Fan [24]	2012	retrospective study	17	51.7	8	9	5	0	17	NR	NR	6	10
Chaoqun Tian [20]	2022	retrospective study	56	52.6	30	26	16	0	54	65.06	NR	71.73	16.5
Lin Han [22]	2016	retrospective study	9	52.25	3	6	1	0	9	NR	NR	48	48
Zhang dao Gong [12]	2016	retrospective study	79	51.8	37	42	23	8	64	74.38	82.86	68.82	24
Yafeng Lyu [11]	2020	retrospective study	124	52.6	54	70	30	0	121	62.88	72.97	69.30	24
Huibing Wang [21]	2021	retrospective study	53	57.75	22	31	9	0	52	NR	NR	120.17	24

### Hearing loss rate and vertigo control rate

**Fig 2:**
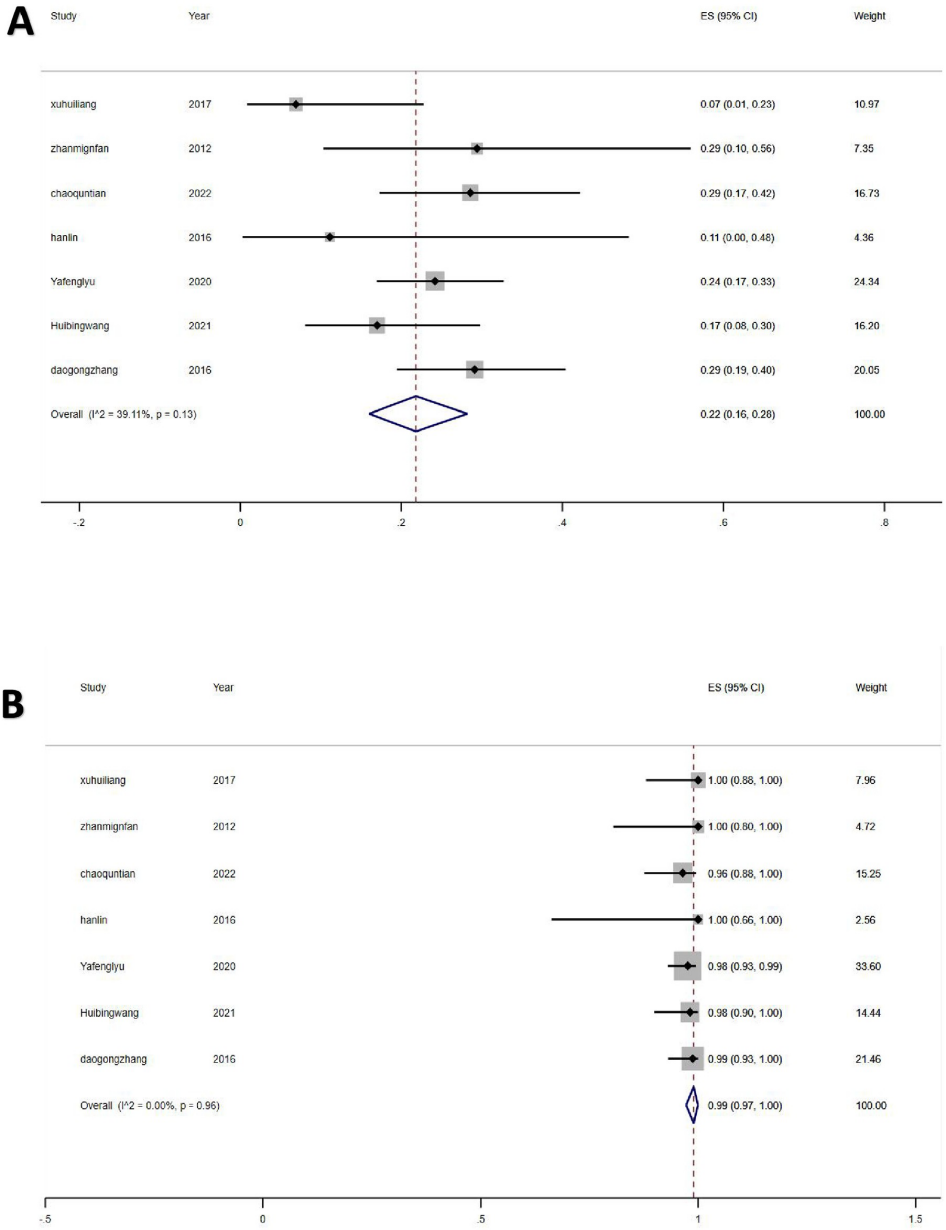
Rates of hearing loss and vertigo control. (A) presents the rate of hearing loss after Triple Semicircular Canal Plugging (TSCP) treatment among patients with Meniere’s disease (MD), derived from a meta-analysis of included studies. (B) It depicts the vertigo control rate after TSCP treatment among MD patients, also based on a meta-analysis.

Seven studies with 367 MD patients were included to analyze the rate of hearing loss after TSCP treatment, of whom 86 had hearing loss after TSCP. In a meta-analysis of the included studies, the rate of hearing loss was 22.0% (95% CI: 0.16–0.28, z = 5.77, p < 0.01) (Fig 2A). These studies had no significant heterogeneity (I^2^ = 39.11%; p = 0.13). As for vertigo control, a total of 360 patients with stage A/B vertigo were well controlled after TSCP. The vertigo control rate of these studies was 99.0% (95% CI: 0.97–1.00, z = 124.22, p = 0.00) and no significant heterogeneity was detected among these studies (I^2^ = 0.00%; p = 0.97) (Fig 2B).

### Subgroup analysis

Further, we conducted a subgroup analysis to explore the effect of duration of disease and follow-up time on hearing loss and vertigo control rates in MD patients after TSCP.

#### Subgroup analysis of hearing loss

**Fig 3:**
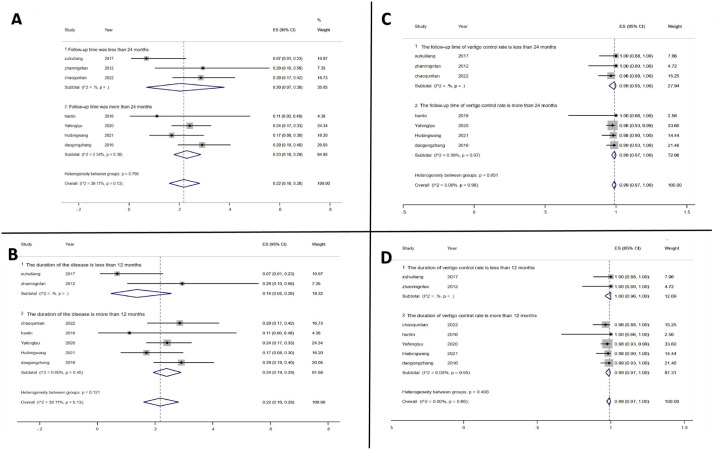
Subgroup analysis. (A) displays the subgroup analysis of hearing loss rates based on follow-up time after TSCP treatment. (B) presents the subgroup analysis of hearing loss rates according to the duration of disease in MD patients. (C) illustrates the subgroup analysis of vertigo control rates based on follow-up time after TSCP treatment. (D) depicts the subgroup analysis of vertigo control rates according to the duration of disease in MD patients.

*Follow-up time*. The length of follow-up time may cause hearing fluctuation in MD patients. Of the included studies, the mean follow-up time of 3 studies was less than 24 months and that of 4 studies was more than 24 months. Subgroup analysis showed that studies with follow-up time less than or more than 24 months had hearing loss rates of 20% (95% CI: 0.07, 0.38) and 23% (95% CI: 0.18, 0.29), respectively. The inter-group heterogeneity test showed no significant heterogeneity between subgroups (p = 0.756) (Fig 3A).

*Duration of disease*. The duration of the disease may be associated with impaired vestibular and cochlear function of the seven studies, two studies had a mean duration of disease of less than 12 months and that of five studies was greater than 12 months. Subgroup analysis showed that the rate of hearing loss in those with duration time less than or more than 12 months was 14% (95% CI: 0.05, 0.26) and 24% (95% CI: 0.19, 0.29), respectively. No significant inter-group heterogeneity was detected between subgroups (p = 0.121) (Fig 3B).

#### Subgroup analysis of vertigo control

*Follow-up time*. To explore whether extended follow-up time would lead to a decrease in vertigo control rate, we conducted a subgroup analysis based on the follow-up time of MD patients. Of the 7 studies included, 3 had a mean follow-up time of less than 24 months, and 4 had a mean follow-up time of more than 24 months. Subgroup analysis showed a vertigo control rate of 99% (95% CI: 0.95, 1.00) in studies with follow-up less than 24 months and that of 99% (95% CI: 0.97, 1.00) in studies with follow-up longer than 24 months. The inter-group heterogeneity test showed no significant heterogeneity among all groups (p = 0.951) (Fig 3C).

*Duration of disease*. MD affected vestibular function if the duration of the disease was long, so the duration of the disease was analyzed by subgroup analysis. Of the seven studies, 2 had a mean duration of disease of less than 12 months and 5 had that of more than 12 months. Subgroup analysis showed that the vertigo control rate in those with a duration time less than or more than 12 months was 100% (95% CI: 0.96, 1.00) and 99% (95% CI: 0.97, 1.00), respectively. The inter-group heterogeneity test showed that there was no significant heterogeneity among the groups (p = 0.408) (Fig 3D).

### Sensitivity analysis and publication bias

#### Sensitivity analysis

Sensitivity analysis was performed on the included studies to further investigate potential heterogeneity in this meta-analysis. The results indicated that no single article has greatly interfered with the analytic results of hearing loss or vertigo control rates. Therefore, our results have good reliability (S1 Fig).

#### Publication bias

The funnel plot was drawn to investigate whether there was publication bias in the included studies. Results of Egger’s test of the symmetry of the funnel plot revealed that the funnel plot of hearing loss (p = 0.947) and vertigo control rates (p = 0.273) was symmetrical (S2 and S3 Figs). Therefore, no underlying publication bias was detected when analyzing hearing loss or vertigo control rates in this meta-analysis (S4 and S5 Figs).

## Discussion

Meniere’s disease (MD) is one of the most common vertigo disorders, characterized by its prevalence in adults aged 40–60 years [25]. The distinctive feature of this disease is the fluctuation of symptoms, with periods of ups and downs. Vertigo episodes can last from minutes to hours, and positional vertigo may occur between or during episodes [26]. In addition, many patients also experience a feeling of pressure or fullness in the ear, accompanied by different forms of tinnitus [27]. Although the basic pathology of MD was identified as endolymphatic hydrops by autopsy in the 1930s [28], the etiology and mechanism of endolymphatic hydrops remain unclear, leading to confusion and uncertainty in the treatment of the disease. Due to the lack of targeted and systematic treatment, a considerable number of patients inevitably develop from early stage to late stage.

Because the pathogenesis of the disease is not clear, the treatment is mainly aimed at controlling the onset of vertigo and delaying hearing loss. Therefore, the treatment of MD is lack of targeted and systematic, resulting in a considerable number of patients inevitably developing from the early stage to the late stage and requiring surgical treatment. The main purpose of surgical treatment is to control vertigo. Traditional vertigo surgery includes endolymphatic sac surgery, vestibular neurectomy, and labyrinthyectomy [16]. With the exception of endolymphatic sac surgery, the latter two procedures completely destroy vestibular function. Endolymphatic sac surgery is the most popular surgical treatment for MD, recommended by the International Federation of Otolaryngology Societies (IFOS) as a third-line treatment but there is a lack of definitive evidence. Labyrinthectomy can better control vertigo but has a cost of complete hearing loss. Vestibular surgical operation has a higher vertigo control rate, but it may lead to facial dysfunction and intracranial infection.

In recent years, due to the concern about the quality of life of patients after vertigo control, the treatment concept of vertigo surgery has also changed significantly. In addition to the focus on the control of vertigo, there is an increasing emphasis on the preservation of residual vestibular and cochlear function and even the reconstruction of function. For example, recent studies have reported that MD can be treated by TSCP, endolymphatic aqueduct occlusion, vestibular implantation, and labyrinth surgical cochlear implantation [29]. In most patients with advanced MD, although vestibular and auditory functions are severely damaged, they are not completely lost. Residual function is still very important, especially for some patients who develop from unilateral to bilateral disease. Therefore, it is very important to preserve vestibular function as much as possible to maintain the balance of the body. In addition, preservation of vestibular and cochlear end organs provides the basis and opportunity for better treatments that may occur in the future [30].

In recent years, TSCP has been reported as a new treatment for MD [9, 31]. In terms of vertigo control, several studies have shown that TSCP can effectively control vertigo at a rate of 97%-100% in MD, which is consistent with the results of our meta-analysis [11, 12, 20, 32]. In addition, as a new inner ear operation, TSCP also has the advantage of reducing chronic postoperative imbalance or vertigo caused by exercise compared with traditional operations such as vestibular resection or labyrinthectomy. Studies have shown that although vertigo symptoms are relieved in most MD patients receiving labyrinthetic surgery, only 50% of them can return to normal work and life [32]. Moreover, vestibular resection is shown to cause longer delays before returning to work [33]. In addition, patients receiving TSCP have a better chance of preserving residual hearing compared with traditional surgery such as disruptive labyrinthectomy. Therefore, the optimal option for vertigo management and hearing rehabilitation is to use TSCP and cochlear implantation simultaneously in patients who had considerable or complete hearing loss prior to surgery [34]. Moreover, for patients whose hearing is available via using hearing aids, their hearing is worth preserving.

TSCP is a novel treatment for MD that can affect hearing although it doesn’t directly affect the cochlea or the cochlear nerve. It remains unclear how TSCP causes hearing loss, which may caused by exolymphatic leakage, inner ear bleeding, ruptured membranous labyrinth, serous labyrinthitis, infection, etc. An investigation on guinea pigs revealed inflammation and fibrosis close to the TSCP surgery site [35]. Another study on guinea pigs suggested that intraoperative membranous labyrinthia injury could lead to severe hearing loss whereas intraoperative peripheral lymphatic leakage would not affect hearing26. Moreover, the duration of the surgery may be correlated with the severity of the hearing loss. The viability of cochlea may be influenced by a shift in membranous labyrinthian pressure during TSCP [12].

TSCP has the advantage of preserving hearing, vestibular, and cochlear function [13]. The membrane-limiting muscle and the external ventricular lymphatic valve are the separating mechanisms between the upper (vestibular) and lower (auditory) labyrinthae [36], which may account for hearing preservation in some patients after TSCP treatment. However, several studies revealed that although the hearing and vestibular function of MD patients were improved during the follow-up, the inner ear function of some patients remained impaired [11, 37, 38].

Currently, no systematic review or study has been done to reveal the frequency of hearing loss after TSCP surgery. Herein, we included seven studies exploring hearing alteration and vertigo control after TSCP in Chinese MD patients. In these studies, the hearing loss rate ranged from 7% to 36%, and a meta-analysis estimated that patients who underwent TSCP surgery had a hearing loss rate of 22.0% (95% CI: 0.16–0.28). The majority of MD patients have a hearing loss of 10–20 dB. Results of subgroup analysis revealed that the duration of disease and follow-up time had limited effects on postoperative hearing loss. We acknowledge that this meta-analysis has some limitations. The studies included in this meta-analysis are cross-sectional, case-control, and cohort studies but not randomized, which has inherent heterogeneity of the findings. Second, data on hearing loss in Chinese MD patients following TSCP surgery are not sufficient to expand the results to all MD patients. Moreover, the number of studies and samples in this meta-analysis is limited. Hence, a prospective large-scale study is needed to verify our findings.

The study has several limitations that should be acknowledged. Firstly, the included studies primarily focused on Chinese populations with Meniere’s disease, which may limit the generalizability of the findings to other ethnic groups or geographic regions. Variations in genetic factors, disease characteristics, and treatment responses across different populations could impact the applicability of the results beyond the studied population. The study design of the included articles was mainly observational (cross-sectional, case-control, cohort), lacking randomized controlled trials. This variation in study design could introduce biases and limit the ability to establish causality between TSCP and hearing outcomes in MD patients. Furthermore, the number of studies included in the meta-analysis was relatively small, potentially limiting the statistical power and generalizability of the findings. A larger sample size and more studies from diverse populations would enhance the robustness and reliability of the conclusions. Additionally, the duration of follow-up varied across the included studies, ranging from 10 to 60 months. This variation in follow-up duration could impact the assessment of long-term outcomes following TSCP treatment and might contribute to heterogeneity in the reported results. Moreover, the study did not extensively explore potential confounding factors or patient characteristics (such as comorbidities, severity of MD symptoms, and surgical techniques) that could influence hearing outcomes following TSCP surgery. The lack of detailed information on these factors may limit the ability to draw definitive conclusions about the effects of TSCP on hearing preservation. The meta-analysis focused primarily on hearing outcomes and vertigo control rates after TSCP treatment. Other important outcomes, such as quality of life measures, patient-reported outcomes, or adverse events related to TSCP, were not comprehensively assessed in this study. The study provides valuable insights into the effects of TSCP on hearing outcomes in MD patients, these findings should be interpreted with caution due to the identified limitations. Future research should aim to address these limitations by conducting well-designed RCTs with larger, more diverse populations and longer follow-up durations to better understand the efficacy and safety of TSCP as a treatment for MD.

## Conclusions

Our meta-analysis of seven studies explored the hearing loss and vertigo control rates after TSCP treatment in MD patients, which indicated that TSCP was efficient in controlling vertigo but with a 22% risk of hearing loss and a pure tone threshold drop of approximately 10–20 dB. In MD patients who have failed to control the symptoms by conventional treatment or non-destructive surgery such as an endolymphatic capsule, TSCP can not only effectively relieve vertigo, but also preserve residual hearing in most patients. If there exists hearing loss, it can be treated by using hearing aids or cochlear implantation. Meanwhile, TSCP may lead to partial hearing loss in stage I and II MD patients, therefore, it should be comprehensively considered when scheduling TSCP. As for the safety of TSCP, no complication has been reported in the included studies. However, the results of this analysis have some limitations, so caution should be exercised in interpreting our conclusions, and more randomized trials are needed to validate our findings.

## Supporting information

S1 Table PRISMA checklist (PRISMA 2020 main checklist and PRIMSA abstract checklist). (PDF)

S2 Table NOS scale scores for the quality of studies included in the systematic review and meta-analysis. (PDF)

S1 Fig Sensitivity analysis of hearing loss and vertigo control rate. (TIF)

S2 Fig Funnel plot of publication bias in studies of the rate of hearing loss after TSCP treatment. (TIF)

S3 Fig Funnel plot of publication bias in studies of vertigo control rates after TSCP treatment. (TIF)

S4 Fig Egger’s test of the rate of hearing loss after TSCP treatment. (TIF)

S5 Fig Egger test of vertigo control rate after TSCP treatment. (TIF)

S1 File Literature retrieval strategy. (PDF)

S2 File All original studies retrieved. (ZIP)

S3 File Original data of the included studies. (XLSX)

S4 File Original data for quality assessment. (ZIP)

## Abbreviations

AO-HNS: American Academy of Otolaryngology-Head and Neck Surgery
CNKI: Chinese National Knowledge Infrastructure Database
ESS: Endolym phatic sac surgery
I^2^: Heterogeneity
IFOS: International Federation of Otolaryngology Societies
MD: Meniere’s disease
NOS: The Newcas tle-Ottawa Scale
PTA: Pure tone audiometry
SCP: Semicircular canal plugging
TSCP: triple semicircular canal plugging
VIP: Chinese Science and Technology Journal Database
