# Supplementary material for: Effects of triple semicircular canal plugging on hearing in patients with Meniere’s disease: A systematic review and meta-analysis
Source: PLoS One. 2024 Dec 5;19(12):e0314348. doi: 10.1371/journal.pone.0314348 (PMC11620686; doi:10.1371/journal.pone.0314348)
Supplement: S2 Table — (PDF) [file pone.0314348.s002.pdf]

S2 Table.NOS Scale scores for the quality of studies included in the systematic review and meta-analysis.

author	years	Case selection				comparability	Result evaluation	Whether the follow-up time was sufficient	follow-up integrality	score
		1	2	3	4					
Xuhui liang[20]	2017	*		*	*		*	*	*	6
Zhaoming fan[21]	2012	*		*	*		*	*	*	6
Chaoqiong tian[17]	2022	*		*	*		*	*	*	6
Gaogong zhang[10]	2016	*		*	*		*	*	*	6
Lin han[19]	2016	*		*	*		*	*	*	6
Yafeng lyu[9]	2015	*		*	*		*	*	*	6
Huibin wang[18]	2021	*		*	*		*	*		5

Note: 1. Case representativeness; 2. Selection of non-exposed cases; 3. Determination of exposure; 4. No outcome event occurs before the study begin.
