# Supplementary figures and images for: Effects of triple semicircular canal plugging on hearing in patients with Meniere’s disease: A systematic review and meta-analysis
Source: PLoS One. 2024 Dec 5;19(12):e0314348. doi: 10.1371/journal.pone.0314348 (PMC11620686; doi:10.1371/journal.pone.0314348)

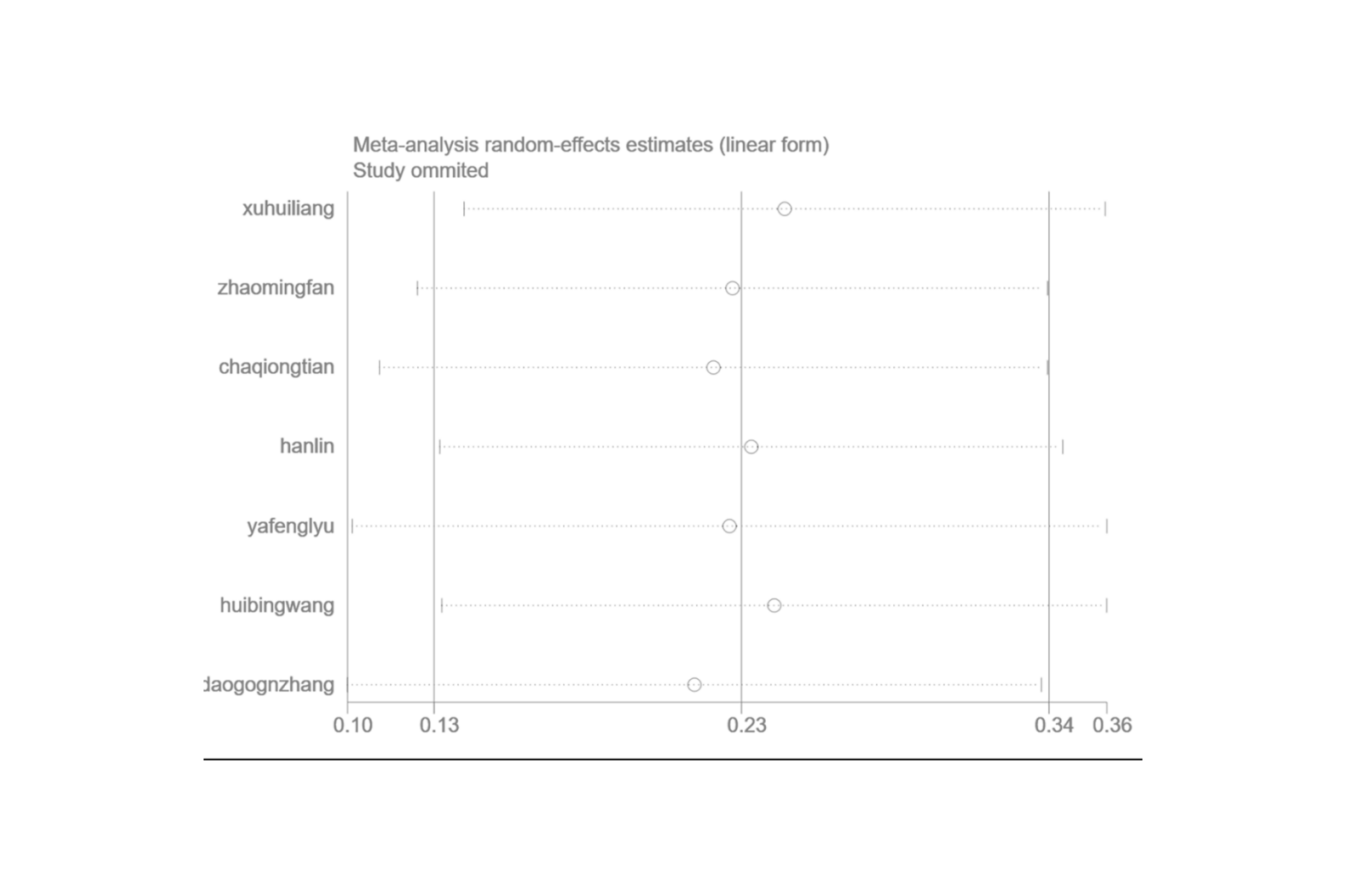

Supplement: S1 Fig — (TIF) [file pone.0314348.s003.tif]

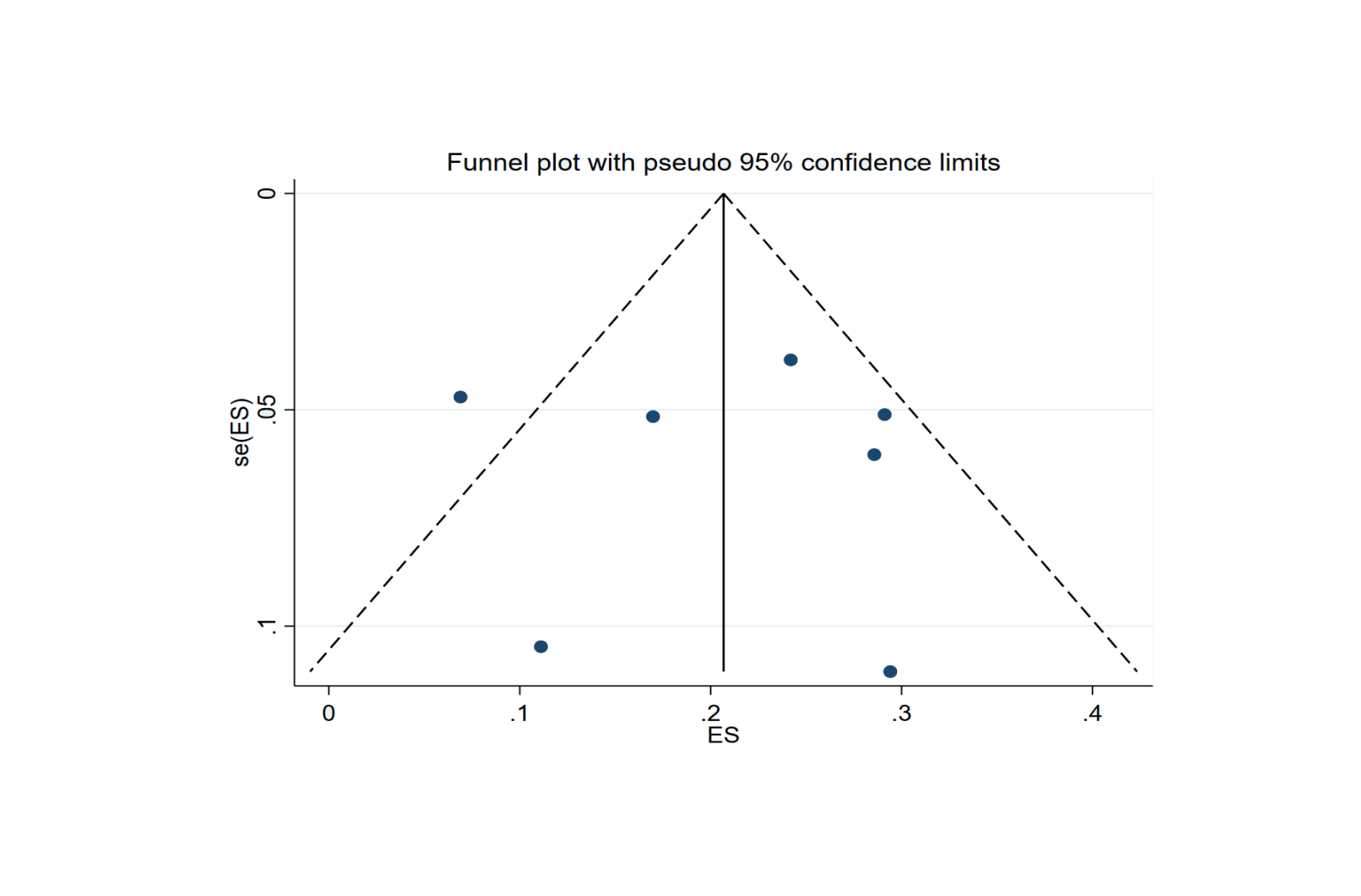

Supplement: S2 Fig — (TIF) [file pone.0314348.s004.tif]

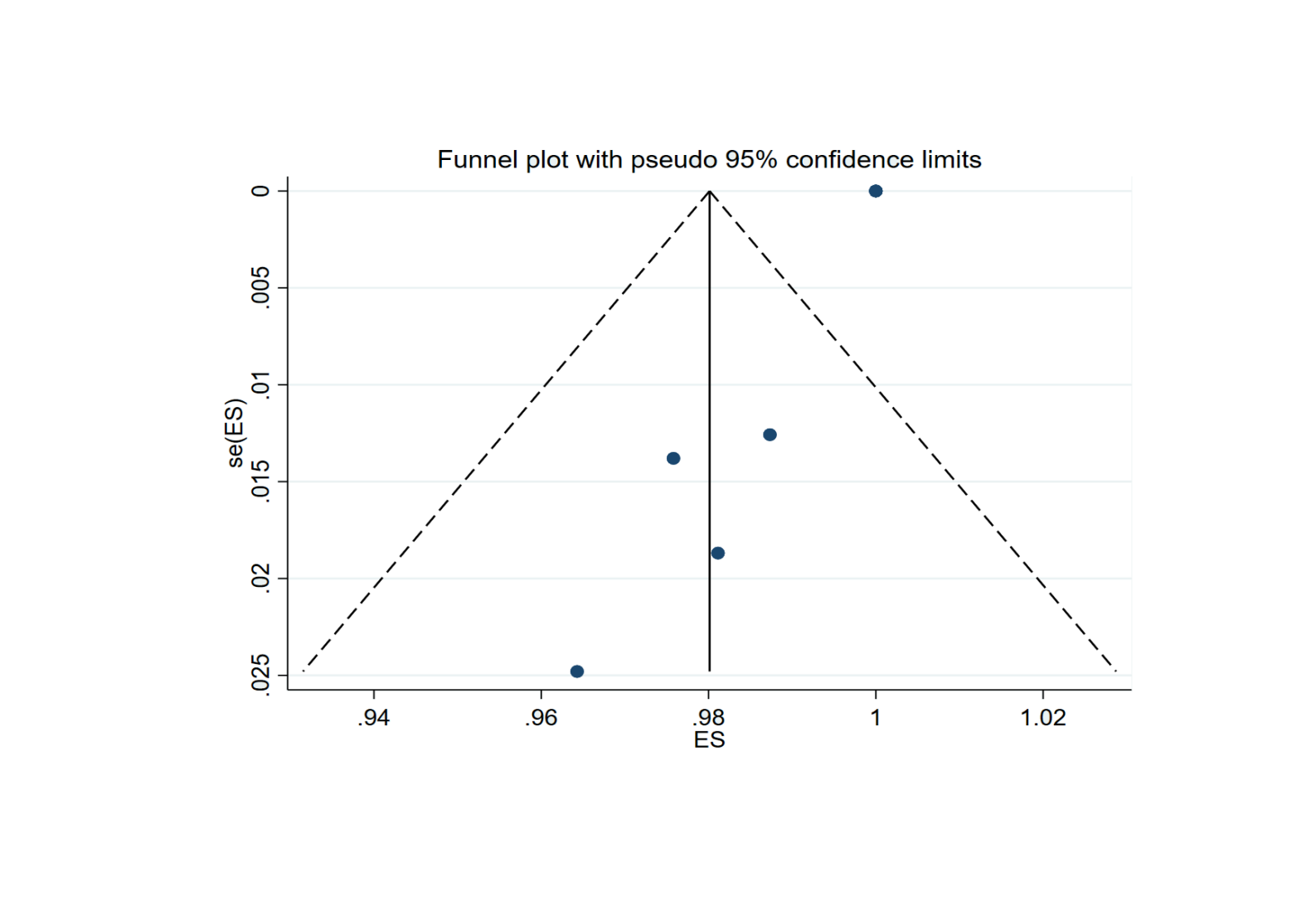

Supplement: S3 Fig — (TIF) [file pone.0314348.s005.tif]

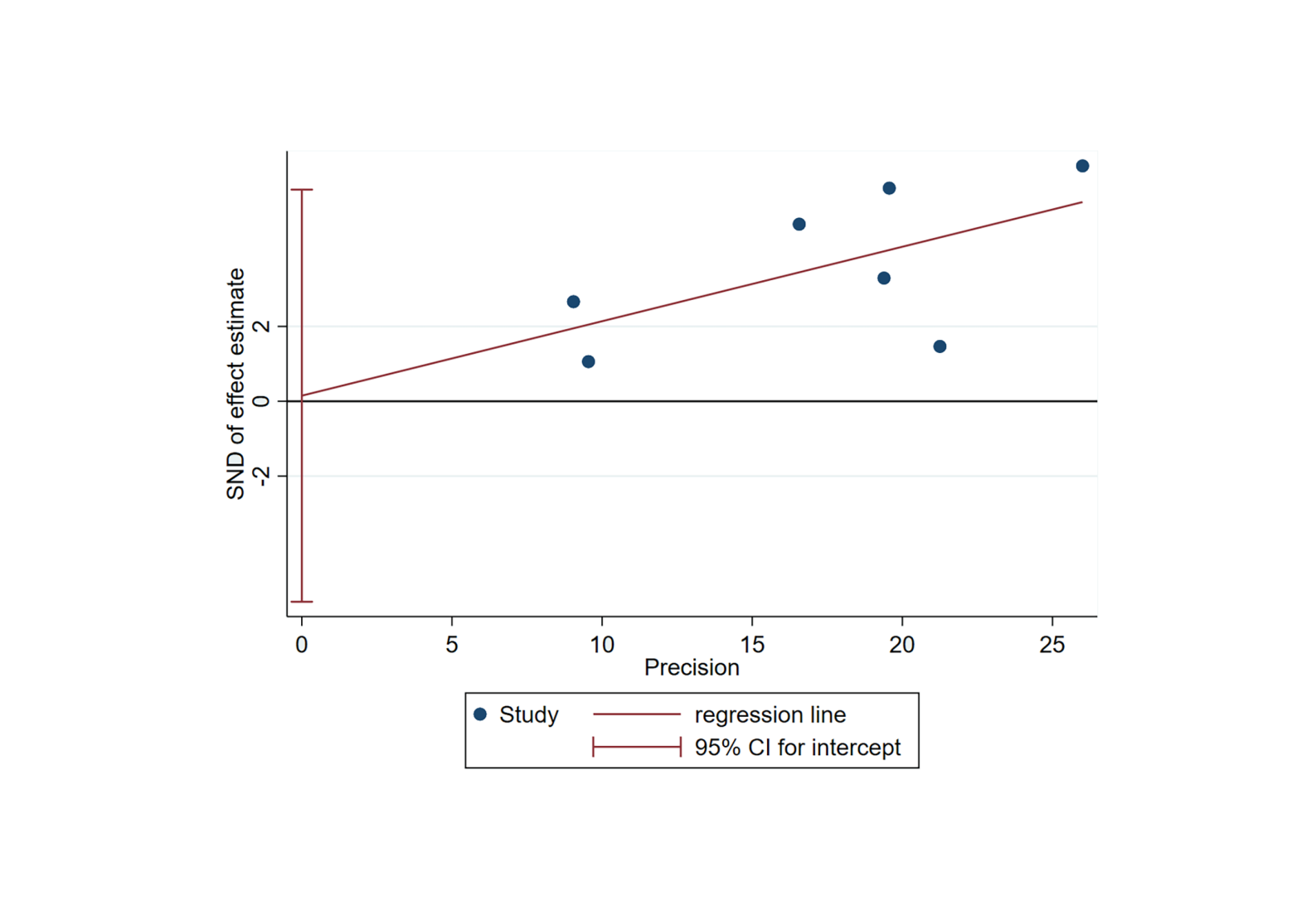

Supplement: S4 Fig — (TIF) [file pone.0314348.s006.tif]

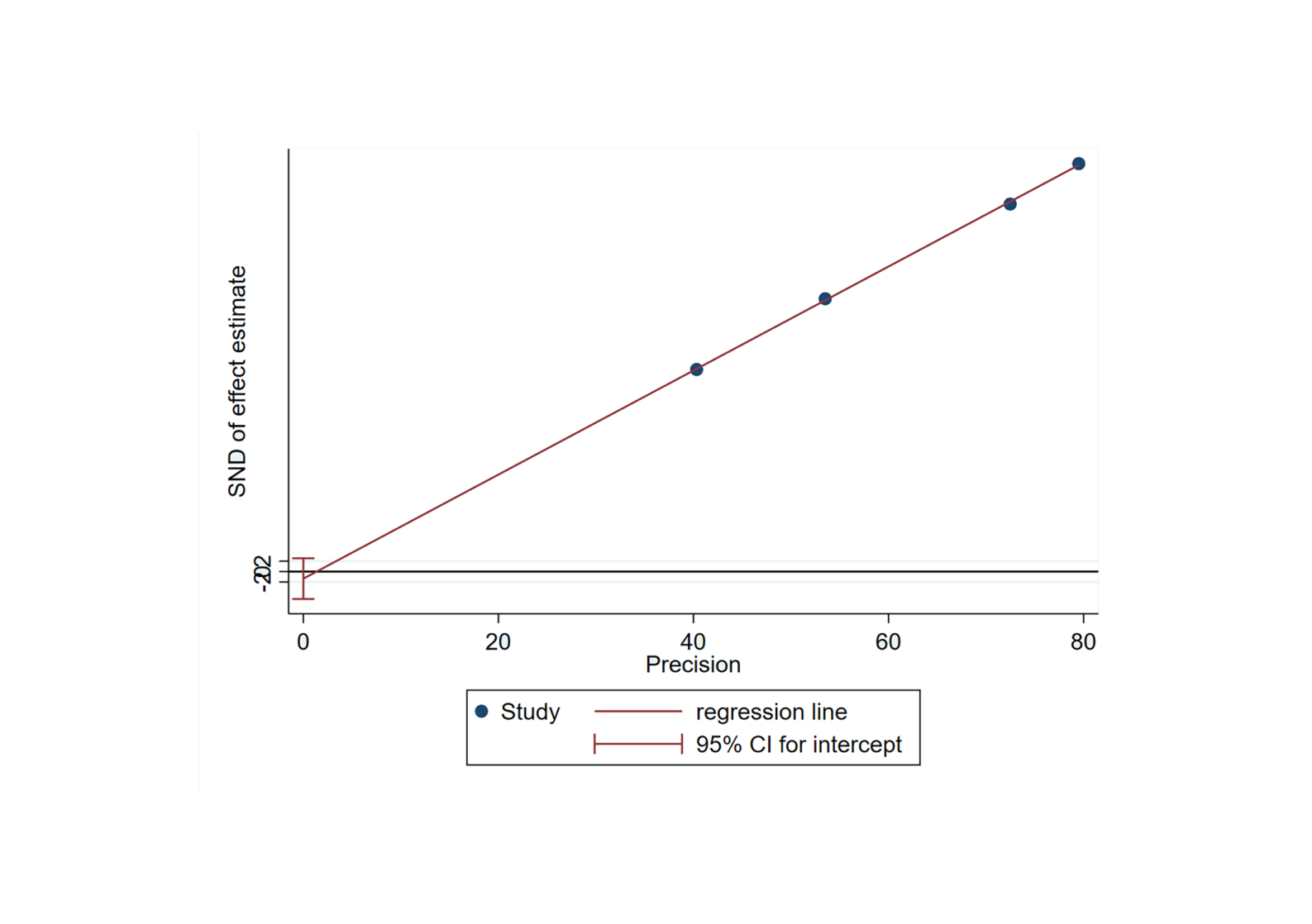

Supplement: S5 Fig — (TIF) [file pone.0314348.s007.tif]
