# Supplementary material for: Effects of triple semicircular canal plugging on hearing in patients with Meniere’s disease: A systematic review and meta-analysis
Source: PLoS One. 2024 Dec 5;19(12):e0314348. doi: 10.1371/journal.pone.0314348 (PMC11620686; doi:10.1371/journal.pone.0314348)
Supplement: S1 File — (PDF) [file pone.0314348.s008.pdf]

S1 File. Literature retrieval strategy

According to the topic of the article, it can be divided into P: Meniere's disease, I: semicircular canal OR S: Case-control studies, randomized controlled studies, cross-sectional studies, cohort studies, case-control and non-randomized controlled studies. Use subject words + free words for literature search. Among them, subject words are connected by AND, AND free words are searched by OR connection, namely P (subject words OR free words) AND I (subject words OR free words) and S (Case-control studies, randomized controlled studies, cross-sectional studies, cohort studies, case-control and non-randomized controlled studies.). Subject words and free words are as follows.

P:Subject word：Meniere，s disease

Free word：Disease, Meniere OR Meniere's Syndrome OR Meniere Syndrome；Menieres Syndrome OR Syndrome, Meniere's OR Ménière's Disease OR Disease, Ménière's OR Diseases, Ménière's OR Ménière's Diseases OR Ménières Disease OR Meniere's Disease OR Disease, Meniere's OR Menieres Disease OR Ménière Disease；Disease, Ménière OR Diseases, Ménière OR Ménière Diseases OR Vertigo, Aural OR Aural Vertigo OR Otogenic Vertigo OR Otogenic Vertigos OR Vertigo, Otogenic OR Vertigos, Otogenic OR Auditory Vertigo OR Auditory Vertigos OR Vertigo, Auditory OR Vertigos, Auditory OR Ménière's Vertigo OR Ménière Vertigo OR Ménière's Vertigos OR Ménières Vertigo OR Vertigo, Ménière's OR Vertigos, Ménière's OR cochlea hydrops OR endolymphatic hydrops OR endolymphatic sac hydrops OR hydrops labyrinthi OR labyrinth hydrops OR labyrinthal syndrome OR meniere disease syndrome OR meniere syndrome OR Meniere's disease OR Menieres disease

OR paroxysmal labyrinthine vertigo OR vertigo, paroxysmal labyrinthine OR Ménière Vertigo OR Ménière's Vertigos OR Vertigo, Ménière's OR Vertigos, Ménière's OR Ménières Vertigo OR Ménière's Vertigo OR Disease, Ménière OR Menieres Syndrome OR Ménières Disease OR Ménière Diseases OR Disease, Meniere's OR Ménière Disease OR Meniere Syndrome OR Ménière's Diseases OR Disease, Meniere OR Ménière's Disease OR Syndrome, Meniere's OR Meniere's Syndrome OR Disease, Ménière's OR Meniere's Disease OR Menieres Disease OR Diseases, Ménière OR Diseases, Ménière's OR Vertigo, Aural OR Auditory Vertigo OR Auditory Vertigos OR Vertigos, Auditory OR Vertigo, Otogenic OR Vertigos, Otogenic OR Otogenic Vertigos OR Otogenic Vertigo OR Aural Vertigo OR Vertigo, Auditory.

I:Subject word： semicircular canal plugging

Free word： horizontal semicircular canal plugging OR semicircular canals plugging OR semicircular canal plugging OR semicircular ducts plugging OR semicircular duct plugging OR Canal, Semicircular plugging OR Semicircular Canal plugging OR Semi-Circular Canals plugging OR Canal, Semi-Circular plugging OR Semi Circular Canals plugging OR Semi-Circular Canal plugging OR horizontal semicircular canal obstruction OR semicircular canals obstruction OR semicircular canal obstruction OR semicircular ducts obstruction OR semicircular duct obstruction OR Canal, Semicircular obstruction OR Semicircular Canal obstruction OR Semi-Circular Canals obstruction OR Canal, Semi-Circular obstruction OR Semi Circular Canals obstruction OR Semi-Circular Canal obstruction.

S: Case-control studies OR randomized controlled studies OR cross-sectional studies OR cohort studies OR case-control OR non-randomized controlled studies.
